# Metrics of Genomic Complexity in the Evolution of Bacterial Endosymbiosis

**DOI:** 10.3390/biology14040338

**Published:** 2025-03-25

**Authors:** Pablo Román-Escrivá, Moisès Bernabeu, Eleonora Paganin, Wladimiro Díaz-Villanueva, Miguel Verdú, José L. Oliver, Vicente Arnau, Andrés Moya

**Affiliations:** 1 Institute for Integrative Systems Biology (I2SysBio), Spanish National Research Council (CSIC), University of València, 46980 València, Spain; pablo.roman@uv.es (P.R.-E.); moises.bernabeu@bsc.es (M.B.); eleonora.paganin@studio.unibo.it (E.P.); wladimiro.diaz@uv.es (W.D.-V.); vicente.arnau@uv.es (V.A.); 2 Barcelona Supercomputing Center (BSC), 08034 Barcelona, Spain; 3 Biomedical Research Networking Center for Epidemiology and Public Health (CIBERESP), 28029 Madrid, Spain; 4 Area of Genomics and Health, Foundation for the Promotion of Sanitary and Biomedical Research of València Region (FISABIO), 46020 València, Spain; 5 Centro de Investigaciones Sobre Desertificación, Consejo Superior de Investigaciones Científicas (CSIC), University of València, Generalitat Valenciana, 46113 València, Spain; miguel.verdu@ext.uv.es; 6 Department of Genetics, Faculty of Sciences, University of Granada, 18071 Granada, Spain; oliver@ugr.es

**Keywords:** genomic complexity, endosymbiosis, complexity metrics

## Abstract

**Simple Summary:**

The increasing biological complexity in evolution poses a challenge in evolutionary biology due to the difficulty of defining and measuring such organism complexity. This can be approximated in several ways, for example, by determining the number of parts that compose it, by the degree of organization of its hierarchical levels of organization, and by assessing the informational content of its genome. In this work, we approached organism complexity via the latter approach using two metrics: Genomic Signature (*GS*) and Biobit (*BB*). We determined the values of these metrics in endosymbiont bacteria versus phylogenetically related free-living bacteria. Endosymbiont bacteria suffer from a process of genetic erosion and the degradation of their genome that would define them, a priori, as less complex than their free-living relatives. We verified that the *GS* and *BB* metrics show lower values in endosymbionts than in their free-living relatives, giving support to the thesis that these metrics reflect the genomic (and biological) complexity of the organisms.

**Abstract:**

Endosymbiosis can be considered a regressive or degenerative evolutionary process characterized at the genomic level by genome erosion and degeneration due to high mutational pressure toward AT (adenine and thymine) bases. The genomic and biological complexity of endosymbionts must be lower than that of the free-living bacteria from which they evolved. In the present work, we contrasted whether two proposed metrics for measuring genomic complexity in both types of bacteria, *GS* and *BB*, reflect their complexity, expecting higher values in free-living bacteria than in endosymbionts. On the other hand, we endeavored to delve into the factors that contribute to the reduction in metric values in endosymbionts, as well as their eventual relationship with six genomic parameters associated with functionality. This study aimed to test the robustness of these proposed metrics in a well-known biological scenario, such as the endosymbiosis process.

## 1. Introduction

The trend toward increasing biological complexity in biological evolution is one of the most challenging issues in evolutionary biology, due in part to the lack of a comprehensive definition of “biological complexity” and its measurement and universality. For instance, we can state that an organism is more complex than another if it has more parts, a higher hierarchical organization, or higher informational content in the genome [1,2,3,4], and we approach biological complexity by referring to the latter. Assuming the genome is the information unit of living organisms and a registry of the evolutionary history inherited over generations, it can indicate the organism’s biological complexity [1,5,6,7,8,9].

A hypothetical approach to characterizing genome complexity is to use metrics that can generally indicate the amount of information in a genome [5,9,10]. In this study, we use two metrics: Genomic Signature (*GS*) and Biobit (*BB*). *GS* is a *k*-mer-based metric summarizing the *k*-mer overrepresentation regarding its expected value [9,11]. *BB* is a genome metric that combines the genome’s entropic and anti-entropic components, considering its *k*-mer entropy [9,12]. Although both metrics depend on the distribution of the frequency table of the *k*-mers present in the genome, they are based on different *k*-mer sizes and theoretical approaches, making the analysis of both metrics complementary. Both metrics analyze genome complexity via different approaches to determine how much a genome differs from a random genome.

The evolution of endosymbiosis may be an excellent context to test whether genome complexity metrics do indeed reflect biological complexity. Endosymbiosis leads to gene loss, a reduction in genome size, and increasing randomness [13,14], so it should be expected that the genomes of endosymbionts would have different metrics to the free-living bacteria from which they are evolutionarily derived. From a functional point of view, what is observed in the evolution of endosymbiosis is a systematic loss of genes until entities are left with minimal genomes [13,14]. When a symbiotic relation is established, the first genes to be lost are those related to mobility and those involved in metabolic pathways for products that can be acquired from the host, and the symbiont has transporters for them [14,15]; from this moment on, a cascade of genome reduction ensues. The process of evolution toward endosymbiosis might be an excellent context for studying the behavior of any complexity measure, including genomic complexity metrics. We expect these measures to yield lower values as endosymbionts become more extreme than those in the free-living bacteria they originate from. Previously, Moya et al. [9] used the *GS* and *BB* metrics in cyanobacterial genomes, concluding that both metrics could measure complexity. However, there was a lack of understanding of these measures in a regressive event. This study tested whether *GS* and *BB* metrics change their values in bacterial endosymbionts to free-living bacteria, thus supporting the genome metric complexity hypothesis.

## 2. Materials and Methods

### 2.1. Genome Set and Species Phylogeny

**Figure 1:**
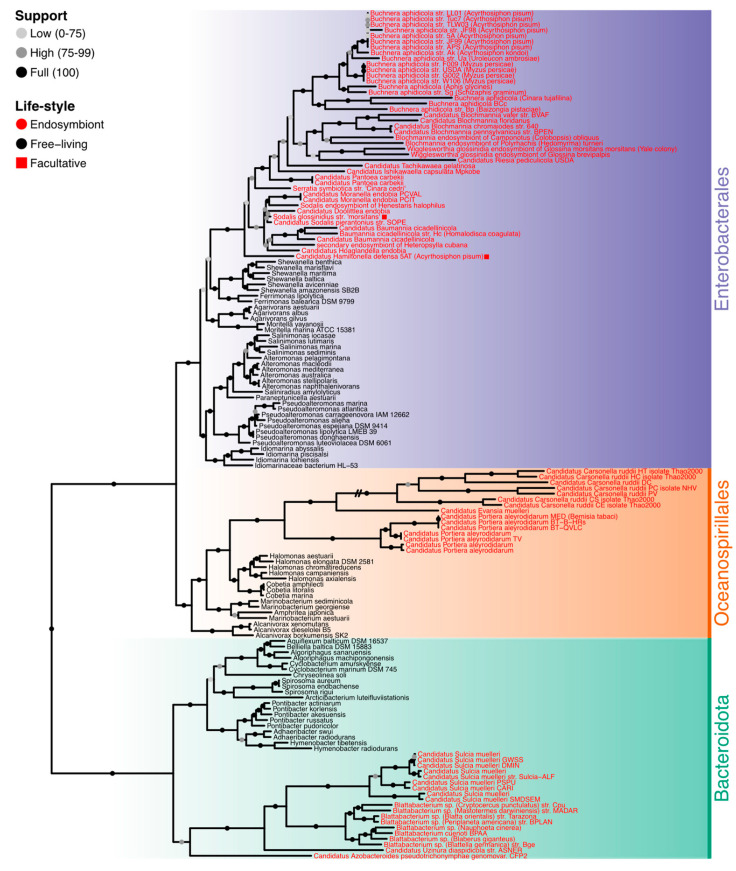
The phylogenetic tree used a concatenated alignment of 27 conserved protein domains. It was inferred using IQ-TREE v2.1.3 under the LG + F + I + G4 model with 4000 ultrafast bootstrap replicates. Dots in branches show support values according to the legend. Species whose names are colored red are endosymbionts, and those in black are free-living. The endosymbionts marked with a red square are facultative, while the rest are obligate.

We selected 78 fully sequenced genomes from endosymbiont organisms of three bacterial clades: Bacteroidota, Oceanospirillales, and Enterobacterales. To compare them and assess the trend to the endosymbiosis analyses, we added 72 free-living bacteria to root each of these clades of endosymbionts. We differentiate both lifestyles with the keyword ‘habitat’. The 19 endosymbiont Bacteroidetes were rooted with 20 free-living Cytophagales species, 15 endosymbionts of Oceanospirillales species were rooted with 15 free-living species from the same clade, and finally, we rooted the 44 Enterobacterales with 37 free-living Alteromonadales. In that last case, we used fewer free-living species due to the sequencing bias toward pathogens and parasites and the assembly quality. To root the tree, we used seven Fusobacteria species (Tables S1 and S2). Maximum likelihood phylogenetic trees using a concatenated alignment of *16S* and *23S* rRNA genes (3981 positions) and 27 conserved proteins (alignment supermatrix with 4102 positions) are shown in Figure S1 and Figure 1. The rRNA tree scored a mean support of 95.04% based on ultrafast bootstrap with 4000 replicates [16], whereas the 27 ribosomal proteins supermatrix tree scored a 95.44% mean support.

We reconstructed two trees: one from a *16S* and *23S* rRNA sequence supermatrix, and another from a supermatrix comprising a set of 27 conserved protein domains. The rRNAs were retrieved directly from the genomes using barrnap v0.9 “https://github.com/tseemann/barrnap (accessed on 17 March 2025)”. Barrnap did not detect four *16S* sequences and one *23S* sequence. These sequences were manually extracted from the annotation files. To reconstruct a protein supermatrix, we downloaded the alignment profiles from the PFAM database and performed hidden Markov model (HMM) searches [17] over all the protein sets of the studied genomes downloaded from the Genome Taxonomy Database (GTDB) [18]. Finally, we obtained those proteins covering more than 75% of species (more than 150 species) and manually removed the possible paralogous sequences (Table S3). We independently aligned the three rRNAs and conserved protein domain sequences using MAFFT-L-INS-i v7.490 [19]. We then trimmed the aligned sequences with trimAl v1.4.15 [20] using the gappyout option. Once aligned and trimmed, the sequences of each set (rRNAs and protein domains) were concatenated, and they were used to infer the species tree. Phylogenies were inferred using IQ-TREE v2.1.3 [21], and the model was selected using ModelFinder [22]. We restricted the models to two sets: JC, HKY, K2P, GTR, and SYM for the rRNA concatenated alignment, and WAG, JTT, LG, and JTTDCMut for the conserved protein concatenated alignment. The robustness of the trees was assessed with 4000 ultrafast bootstrap replicates [16].

### 2.2. Genomic Metrics and Parameter Calculation

For each genome, we computed two genome metrics: *GS* and *BB* (see the following sections for details). We also retrieved six genomic parameters from the GFF (annotation file that describes DNA, RNA, and protein sequences) files linked to each genome (number of CDSs, number of genes, number of rRNAs, gene mean length, genome length, and GC content) and added the percentage of hapaxes. A hapax is defined as a sequence appearing just once in a genome, or in this case, a *k*-mer with an absolute frequency of 1 in the frequency table of the *k*-mers of a genome, for a given value of *k*. We computed the percentage of hapaxes to the total number of *k*-mers.

### 2.3. Genome Signature (GS)

The GS metric focuses on the *k*-mer content of a given genome [9]. For an alphabet of four characters, as DNA, and defining a word length of *k* characters, we can obtain a maximum of 4*^k^* words (*k*-mers). Then, the expected occurrence value of every *k*-mer is

$$EV=\left(\sum_{j=1}^{4^{k}}n_{j}/4^{k}\right),$$

where $n_{j}$ is the total number of *k*-mers found in the genome. For a specific value of *k*, a value of *GS* (*GS_k_*) can be calculated as

$${GS}_{k}=\frac{1}{\sum_{j=1}^{4^{k}}n}\cdot \sum_{i=1}^{4^{k}}\left|n_{i}-EV\right|.$$

$n_{i}-EV$ serves as the mean centering, with $n_{i}$ being the number of *k*-mers found for a specific sequence, and the final value is divided by

$$\sum_{j=1}^{4^{k}}n_{j}.$$

In this way, genomes of different sizes can be compared. A random genome of the same size and base composition as the given genome is first created to obtain the optimum value of *k* for a given genome. *GS_k_* is then calculated on the provided genome (*GS_g_*) and the random genome (*GS_r_*), starting from *k* = 2, and *GS_r_* is subtracted from *GS_g_* to remove random noise from the metric to obtain a preliminary value of *GS* (*GS_p_*).

$${GS}_{p}={GS}_{g}-{GS}_{r}.$$

Finally, we repeat the procedure, increasing the value of *k* up to 16. The *GS* value for that genome is the maximum value obtained for *GS_p_.*

### 2.4. Biobit (BB)

The *BB* metric is a logistic map that balances a genome’s entropic and anti-entropic components [12]. *BB* compares the true genome with a random equifrequent one with the same length. First, the *k*-mers yielding the maximum entropy of real and random equifrequent genomes are calculated and compared. The entropy of a genome of length *G* (*E_2L_*_(*G*)_) takes a value between the minimum (log_4_(*G*), denoted *L*(*G*)) and the maximum (2*L*(*G*)). The entropic (*E*(*G*) = E*_2L_*_(*G*)_ − *L*(*G*)) and anti-entropic (*A*(*G*) = 2*L*(*G*) − E_2*L*(*G*)_) components of the genome are then calculated, and then *E*(*G*) + *A*(*G*) = *L*(*G*). These elements can be combined, nonlinearly, by

$$BB\left(G\right)=\sqrt{L(G)}\sqrt{\frac{A(G)}{L(G)}}{\left(1-2\frac{A(G)}{L(G)}\right)}^{3}.$$

### 2.5. Statistical Analyses

We performed a correlation analysis between all the genomic variables and the complexity indexes retrieved. This analysis was performed to assess what variables may be affecting each of the metric values and to evaluate if any of the metrics indicate functionality. Phylogenetically informed correlations were performed using an in-house function with the variance–covariance matrix, calculating Pagel’s lambda [23,24] for each pair of traits calculated using the phytools v2.1-1 [25] R package. This matrix was converted to correlations between traits matrix with the stats R package “https://www.r-project.org/ (accessed on 17 March 2025)”. Finally, *t* values for each correlation value *r* were obtained using the formula $t=r\sqrt{\left(n-2\right)/\left(1-r^{2}\right)}$, and a *p*-value was derived from a *t*-student distribution with *n* − 2 degrees of freedom. The *p*-values were corrected via the Holm–Bonferroni method [26] to control the family-wise error rate (FWER).

We used two-sample comparisons to contrast the differences between free-living and endosymbiont organisms in each variable. We applied phylANOVA [23] to consider the phylogenetic relationships among lineages. The same dataset has also been used to assess a principal component analysis (PCA) to further investigate the effect of and relationship between the genomics variables on and between the complexity indexes and to assess which variables better characterize the differences between the samples. We used a correlation matrix to perform the PCA, as the scales of the variables were very different. The PCA was phylogenetically informed by in-house functions using phytools, calculating Pagel’s lambda for the whole matrix [23,24].

### 2.6. Phylogenetic Signal

Pagel’s lambda was used to phylogenetically inform correlations and PCAs. The phylogenetic signal of each trait in the entire tree and the three clades was analyzed using Blomberg’s *K* [27]. When *K* is significantly different from zero, the trait shows a phylogenetic signal; that is, the trait resembles more in closer species than expected by chance. A robust phylogenetic signal is assumed when *K* > 1 since *K* = 1 is the predicted value under Brownian evolution.

## 3. Results and Discussion

### 3.1. Phylogenetic Analyses

To analyze the endosymbiosis phylogenetic transition, we first selected 150 bacterial genomes, 78 of which are from bacterial endosymbionts and the other 72 from free-living bacteria (Tables S1 and S2). These free-living bacteria draw the evolutionary path to endosymbiosis in three main lineages: Bacteroidota phylum, Enterobacterales, and Oceanospirillales orders. We used seven Fusobacteria species to root the entire tree. As indicated, the transition to endosymbiosis was assessed using free-living relatives for each lineage. As expected, the Oceanospirillales and Enterobacterales form a sister clade of the Bacteroidota. The internal topology of each group differs between rRNA and protein trees. For the rRNA tree (Figure S1), some free-living Oceanospirillales are in the root of Proteobacteria, and others are placed in the root of the Alteromonadales and Enterobacterales clade. Despite this topology, the protein supermatrix tree (Figure 1) resolves these arrangements better, and the three groups are correctly clustered, following previously reported topologies [17,28]. The protein supermatrix tree was thus used for the analysis where a tree was needed.

### 3.2. Phylogenetic Signal

**Figure 2:**
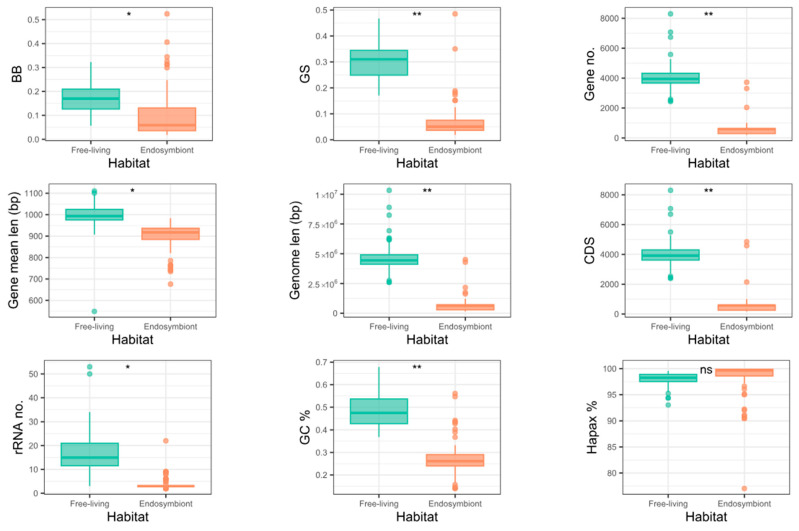
Boxplots of each trait for free-living and endosymbiont genomes in the whole tree. Stars indicate the statistical significance of the mean based on the phylogenetically informed test.

Almost all phylogenetic signals of all the study traits across the whole tree, and in the three clades where endosymbiotic events occurred (Figure S2 and Table S4), were significant. None of the phylogenetic signals were stronger than Brownian evolution (i.e., K > 1) in the whole tree or the Enterobacterales clade. However, some traits with *K* > 1 were found in the Oceanospirillales (number of genes and CDS, genome length, and GC content) and Bacteroidota (GC content and *GS*) clades. Taking Brownian motion as a reference, *K* values greater than 1 indicate more variance among clades than expected by chance. In contrast, values lower than 1 indicate greater variance within clades than expected by chance. When studying the differences in distribution for many of the traits between endosymbionts and free-living organisms (Figure 2), the greater differences seem to correlate with greater values of *K*, which would make sense as the difference among the clades is greater than expected.

### 3.3. Metrics, Genome Parameters, and Phylogenetic Correlations

**Figure 3:**
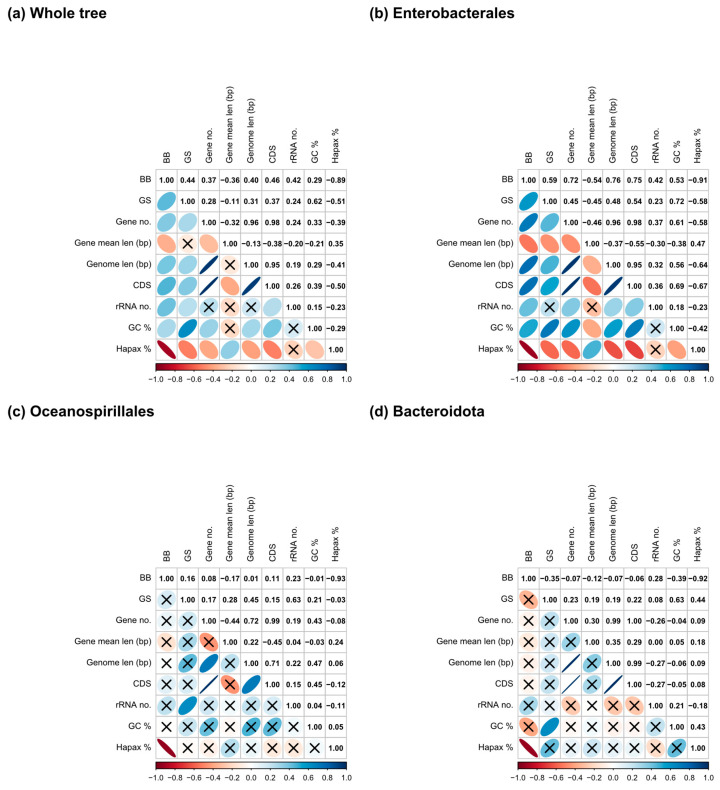
Phylogenetic correlation plots for (**a**) the whole tree, (**b**) the Enterobacterales, (**c**) the Oceanospirillales, and (**d**) the Bacteroidota clades. Crosses indicate statistically non-significant correlation values, and the correlation value is shown in color according to the legend.

Table S5 shows the values of the metrics and parameters calculated (from now on called traits when referring to both). To assess if both metrics indicate functionality, we performed phylogenetic correlation analyses concerning the genome parameters for the whole tree and the three lineages, respectively (Figure 3). As can be observed, the *GS* and GC content show a moderate positive (0.62) relationship in the entire tree (Figure 3a) and in the Bacteroidota clade (0.63) (Figure 3d), becoming stronger in the Enterobacterales clade (0.72) (Figure 3b). For *BB*, the only correlation with an absolute coefficient greater than 0.85 is with the percentage of hapaxes, which holds in every lineage. Nevertheless, for the Bacteroidota and Oceanospirillales (Figure 3c,d), many correlations were not statistically significant, possibly due to the lower number of taxa involved.

### 3.4. Principal Component Analysis of Traits That Discriminate Between Bacteria Lifestyles

**Figure 4:**
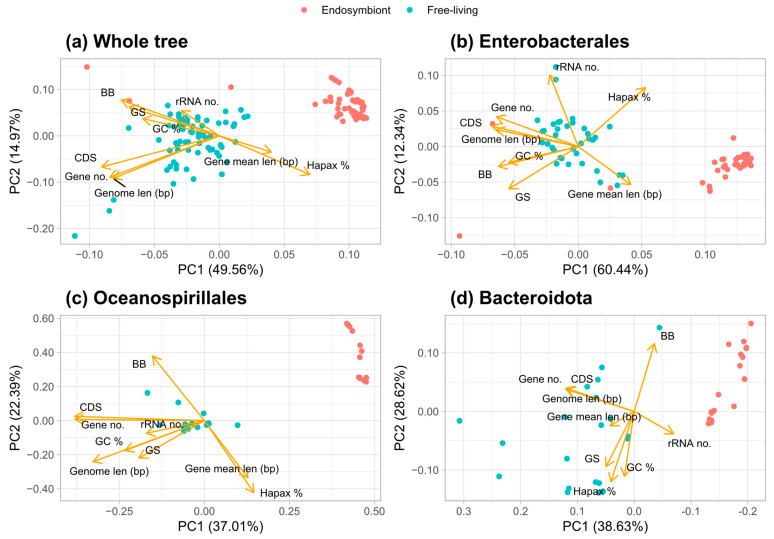
Phylogenetic-informed principal component analysis (PCA) of (**a**) the entire tree, (**b**) Enterobacterales, (**c**) Oceanospirillales, and (**d**) Bacteroidota clade. Arrows show the loadings for each variable, and the points represent the genomes; their color shows the lifestyle of the organisms according to the legend.

The principal component analysis (PCA) of all the traits (metrics and parameters) reveals that these traits discriminate between the habitats of the organisms (Figure 4). For all the bacteria studied (Figure 4a), the first component can almost distinguish between free-living and endosymbiont organisms independently. For the calculation of this component, the more important variables are the number of CDSs, number of genes, genome length, and percentage of hapaxes. For the Enterobacterales lineage (Figure 4b), three clusters are observed, with the free-living organisms being the ones in the center. When analyzing the Oceanospirillales and Bacteroidota lineages, the first component is enough to discriminate between the lifestyles (Figure 4c,d).

### 3.5. Genomic Base Composition Drives the GS and BB Values

**Figure 5:**
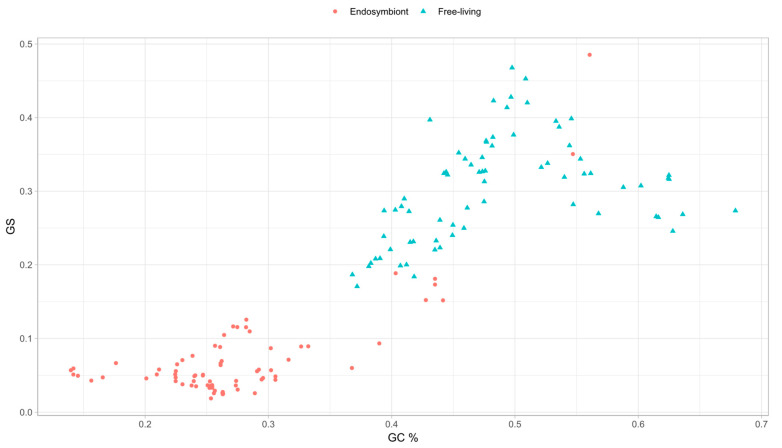
Plot representing the values of Genomic Signature against GC content. Values in red correspond to endosymbionts, while values in blue correspond to the free-living organisms.

From the results of the phylogenetic correlations, regarding the comparison of GC content versus the value of *GS*, we obtained a moderate positive relationship in most of the clades. Analyzing this in detail, we represented the correlation for all the genomes (Figure 5). As can be observed, there is a quadratic correlation pattern where the maximum value of *GS* corresponds to the genomes with a GC content near 50%. When the values of GC content decrease or grow by around 50%, the *GS* metric values always tend to decrease. This decrease in the *GS* metric with extreme GC content values indicates that the metric may be related to the randomness of the genome sequence, as a genome with equiprobable base frequencies is more likely to have an even distribution of *k*-mers. A genome with uniform base frequencies would lead to a more random genome (maximum *GS*) than one with a biased nucleotide base composition, where the probability of specific arrangements of nucleotide bases of length *k* would be higher.

*GS* is a measure based on the overrepresentation and underrepresentation of the *k*-mers. More homogeneous genomes with a high number of nucleotide base pairs, such as A and T in the case of endosymbionts, will thus favor the overrepresentation of AT-rich *k*-mers, which will result in an uneven distribution of the *k*-mer frequency and, thus, in higher $n_{i}$ values. Consequently, $n_{i}$ >> EV produces an increase in *GS_g_* (see the *GS* equation in methods). Otherwise, genomes with equal proportions of nucleotide bases (GC content around 50%) will provide an even *k*-mer distribution, resulting in $n_{i}$ values closer to their EV, approximating the term $\left|n_{i}-EV\right|$ to 0, and leading to lower *GS_g_* values. Why then is the opposite behavior observed in Figure 5? This is due to the effect of *GSr* on the calculation of the metric. In the genomes of the endosymbionts, there is an overrepresentation of AT-rich *k*-mers, and considering that with *GS,* we are working with relatively small *k* values, which makes the frequency tables of the given genome and the random genome quite similar in distribution. The values of *GS_g_* and *GS_r_* are far more similar the further away the given genome is from a 50% GC content. The representation of *GS_r_* and *GS_g_* against GC content is shown in Figure S3, supporting the presented idea. We see this mathematical reasoning in the data where we can observe the peak in Figure 5 occurring strictly at a GC content of 50%. Uneven *k*-mer distributions due to GC content then decrease the *GS* value. This peak is the reason behind the linear correlation values between both traits, but as shown here, they are closely related. When we computed a quadratic regression with the data, we obtained a multiple R-squared value of 0.7707 with an adjusted R-squared values of 0.7676, showing a high quadratic correlation.

**Figure 6:**
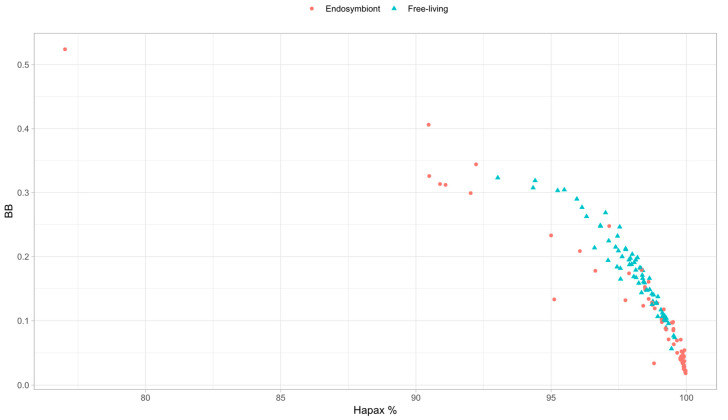
Plot representing the values of *BB* against the percentage of hapaxes. Values in red correspond to endosymbionts, while values in blue correspond to the free-living organisms.

We also observed that the percentage of hapaxes is strongly positively correlated in all the clades. When analyzing *BB*, we then represented the percentage of hapaxes versus the value of *BB* to observe how these unique *k*-mers’ presence may be driving the metric (Figure 6). We can observe a significant negative Pearson correlation of −0.89, so the percentage of hapaxes may influence the metric such that the higher the number of *k*-mers appearing once, the lower the metric value. That is also consistent with the metric principles because, in random genomes, all *k*-mers should be hapaxes [12]. We also observed seven endosymbiont outliers in the *BB* metric (Figure 2), allowing for us to further analyze these organisms’ nature and metrics. It is worth noticing that these outliers tend to present higher or similar values to the free-living bacteria with the highest *BB* value. However, why is *BB* so high in the outliers?

### 3.6. Outlier Analyses of BB

The highest value of *BB* corresponds to Candidatus Sodalis pierantonius str. SOPE, a *Sitophilus oryzae* primary endosymbiont [29]. The genome of this organism presents a high percentage of mobile elements, especially insertion sequences (ISs), typically present at the first stages of the endosymbiont process, as is the case in this endosymbiont. The high percentage of these repetitive mobile elements decreases the percentage of hapaxes, favoring the homogeneity of the genome, and seems to increase the *BB* value.

The next highest value of *BB* corresponds to Candidatus Hamiltonella defensa 5AT, a secondary endosymbiont of *Acyrthosiphon pisum* [30]. This genome is also colonized by mobile elements that constitute around 20% of the genome, leading to a decrease in the percentage of hapaxes and an increase in the metric.

The next organism with a high *BB* value is *Sodalis glossinidius* str. ‘Morsitans’, a secondary endosymbiont of *Glossina morsitans* [31]. In this case, a moderate percentage of ISs (around 2.5% of the genome) was observed, possibly linked to a recent evolutionary link between the endosymbiont and the host [32]. The presence of these sequences decreases the percentage of hapaxes as before, increasing the metric value. In those three previous examples, we observed some of the highest percentages of GC in our endosymbiont dataset (more than 40%) and also some of the largest endosymbiont genomes in our dataset, possibly indicating a recent evolutionary link with their respective hosts.

Candidatus Hamiltonella defensa 5AT and *Sodalis glossinidius* str. ‘Morsitans’ are also the only two facultative endosymbionts in the dataset that reflect the recent acquisition by their hosts. Finally, the last four outliers correspond to the same organism, *Candidatus* Carsonella ruddii, a primary endosymbiont of different species of psyllids [33]. Unlike what we observed with C.H. defensa and *S. glossinidius*, these organisms have the lower percentages of GC in our endosymbiont dataset (around 15%). They are the shorter ones of that dataset (fewer than 160.000 bases). The percentage of hapaxes is lower because this genome seems to retain some specific sequences, leading to the retention of some specific *k*-mers, which decreases the total percentage of hapaxes due to the short size of the genomes. This decrement, as before, seems to increase the *BB* value.

As can be seen, the number of hapaxes is inversely related to *BB*. Moreover, the hapaxes proportion is directly related to the genome heterogeneity; the lower the number of hapaxes, the higher the homogeneity of a genome (as it has more repeated *k*-mers). Conversely, a genome with a high proportion of hapaxes becomes more heterogeneous as fewer repetitive *k*-mers are seen, and we could thus relate *BB* to the *k*-mer heterogeneity.

### 3.7. Genome Complexity and Metrics

To assess the complexity hypothesis, we first studied the statistical difference in each metric between the free-living and endosymbiont lifestyles for each group, informing the tests with the corresponding phylogeny. Some genomic parameters show significantly lower values for the endosymbiont genomes in some of the lineages (Figure S4 and Table S6). These are the number of genes in all the lineages, genome length, and CDSs in Enterobacterales and Oceanospirillales, as well as the GC content in Oceanospirillales. With *GS,* a significant difference can be observed in the whole tree and the Oceanospirillales, while *BB* shows no significantly lower values for the endosymbionts in any clade. This non-significant difference regarding the Enterobacterales is due to the presence of the outliers mentioned above, which manifest as outliers in all the whole tree and Enterobacterales plots. If we remove the seven outliers, there is a significant difference in the *BB* values between the endosymbionts and the free-living organisms in the whole tree (*p*-value 0.03) but not in the Enterobacterales (*p*-value 0.09). For *GS,* removing these outliers reveals a significant difference in the whole tree (*p*-value 0.009), and there is also a significant difference in the Enterobacterales (*p*-value 0.009).

Nevertheless, instead of perceiving the same behavior, *BB* and *GS* do not show remarkably significant differences between endosymbionts and free-living organisms in Bacteroidota, and only *GS* is able to discriminate the two habitats regarding the Oceanospirillales. For *BB,* it may be due to having fewer taxa in those two clades than in the Enterobacterales one. *GS’s* ability to discriminate in the case of the Oceanospirillales may be explained by analyzing the base composition of the genomes (Figure S5). As can be seen, when the difference in the AT content (or GC content) is greater between the habitats, the metric seems to discriminate better between them. With the results obtained, *GS* represents a complexity that is based more on the informational content of the genomes and its relationship with entropy. In contrast, *BB* represents a complexity based the determined adaptability or plasticity of the genome, as observed regarding its outliers in transitional phases of the endosymbiosis process, which are supposed to have been more subjected to a higher dynamism.

For *GS*, the degeneration process characteristic of the endosymbionts brings the genomes to a base composition that deviates more from a 50% GC content, thus decreasing the metric. With *BB*, free-living organisms tend to have a greater metric value, which correlates with the theoretical biological complexity. However, we still observe a higher *BB* value on the genomes of species in a transitional stage due to the content of repetitive structures.

## 4. Conclusions

We demonstrated that *GS* decreased during the endosymbiosis process, and *BB* has significant differences in the whole tree. For *BB*, we explained the Enterobacterales outliers within the context of the first steps of the endosymbiosis process.

We also observed that the selection bias toward AT in endosymbiont genomes is related to the lower values of *GS*. This metric accounts for the representation of the *k*-mers by retrieving their frequency to the expected number of *k*-mers to find. More homogeneous genomes thus have more similar *k*-mers, indicating an overrepresentation of certain *k*-mers. Considering the behavior of the metric when analyzing the random genome, the lower values in the case of the endosymbionts are explained.

We concluded that *GS* may be too limited to the actual contents of the genome and their relationship with the entropy of a random genome. At the same time, *BB* shows more promise as a metric that could quantify a larger part of the complexity of the genome and its adaptability.

More studies investigating other biological scenarios should be performed to establish *GS* and *BB* as universal metrics of genomic complexity as well as their relationship with biological complexity.

## Data Availability

The data presented in this study are available on request from the corresponding author.

## Supplementary Materials

The following supporting information can be downloaded at https://www.mdpi.com/article/10.3390/biology14040338/s1, Figure S1: A phylogenetic tree was performed using a concatenated alignment of *16S* and *23S* rRNA genes. Figure S2: Phylogenetic signal heatmap. Figure S3: Representation of *GS_r_* and *GS_g_* against GC content. Figure S4: Boxplots of each trait for free-living and endosymbiont genomes in each studied clade. Figure S5: Barplots of the mean base composition of the genomes for each habitat and within each group. Table S1: Taxonomy, accession numbers, and FTP links for the used genomes stored in NCBI. Table S2: Summary of the number of endosymbiont and free-living species for each bacterial class and order. Table S3: Table showing the used ribosomal proteins in the phylogenetic tree. Table S4: Summary statistics of the phylogenetic signal analysis. Table S5: Values for the metrics and the genomic features. Table S6: Mean comparisons between the free-living and endosymbiont genomes in Bacteroidota, Oceanospirillales, and Enterobacterales clades.
