# Supplementary material for: Metrics of Genomic Complexity in the Evolution of Bacterial Endosymbiosis
Source: Biology (Basel). 2025 Mar 25;14(4):338. doi: 10.3390/biology14040338 (PMC12024623; doi:10.3390/biology14040338)
Supplement: Supplementary file 1 [file biology-14-00338-s001.zip › Supplementary_Figures.pdf]

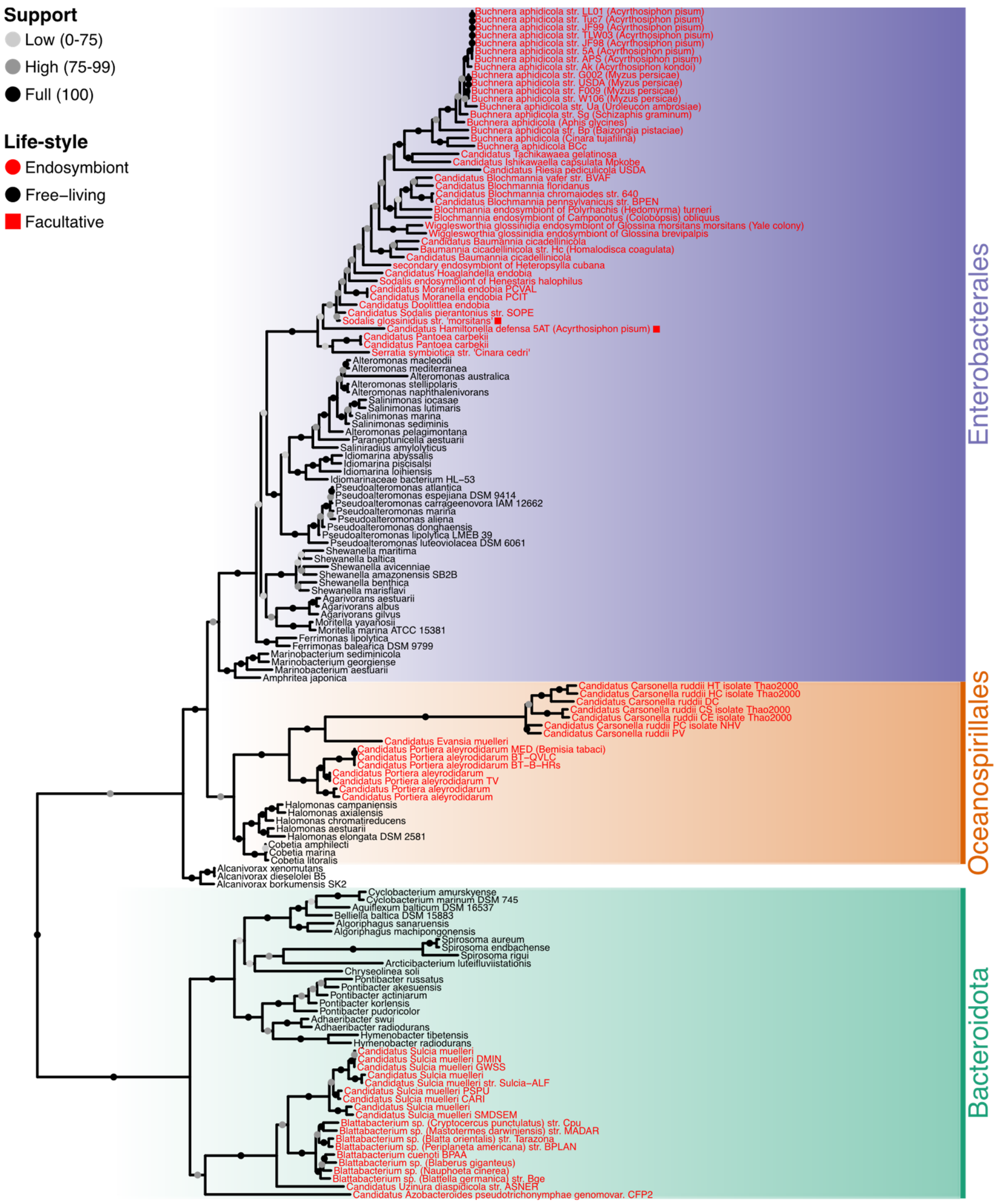

Supplementary Figure S1. The phylogenetic tree used a concatenated alignment of 16S and 23S rRNA genes. It was inferred using IQ-TREE v2.1.3 under the GTR+F+I+G4 model with 4000 ultrafast bootstrap replicates. Dots in branches show the support values according to the legend. Species' names colored in red are endosymbionts, and in black are free-living. The endosymbionts marked with a red square are facultative, while the rest are obligate.

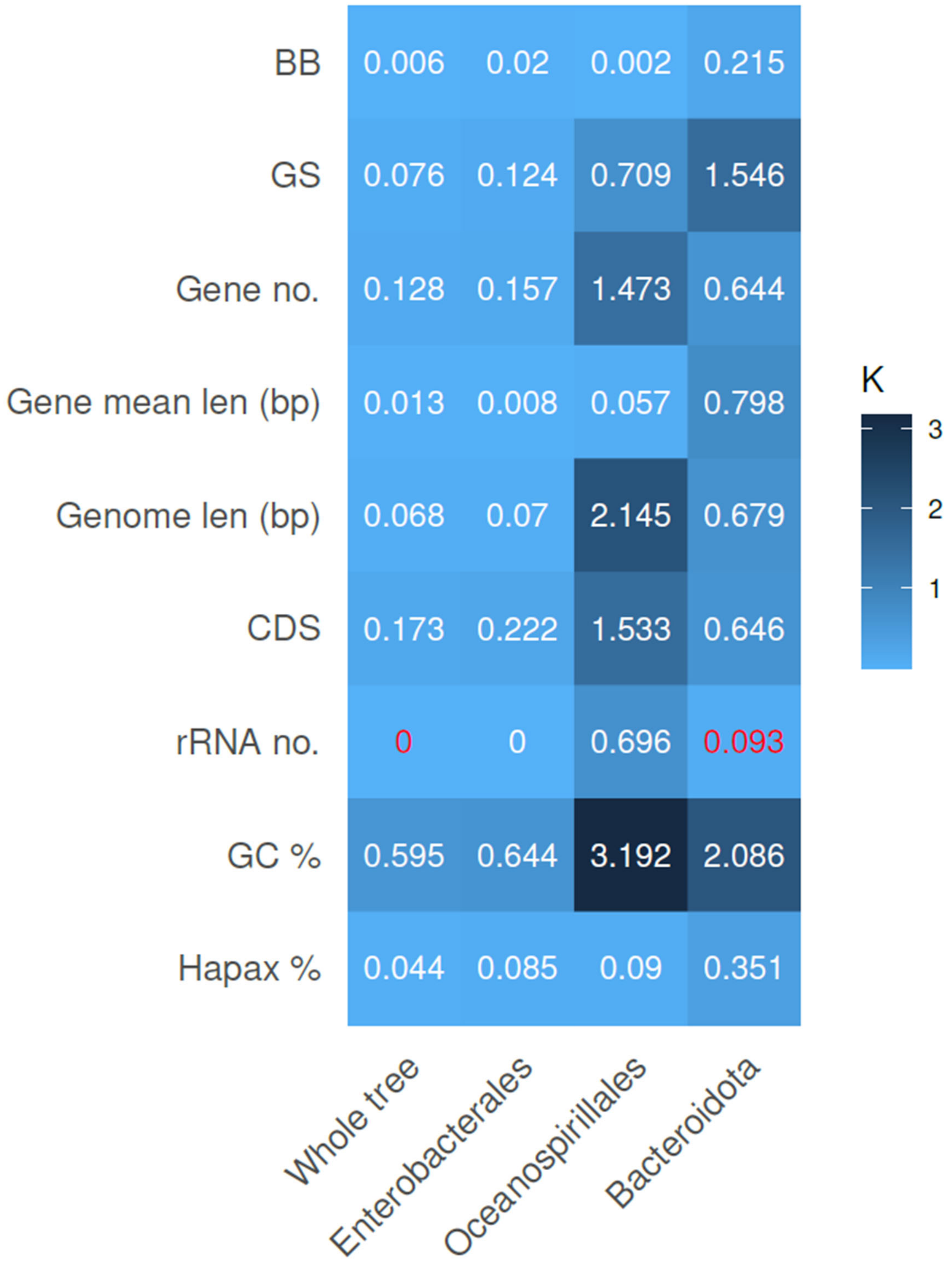

Supplementary Figure S2. Phylogenetic signal heatmap. Values in white show statistically significant Blomberg's *K* values while values in red show not statistically significant values.

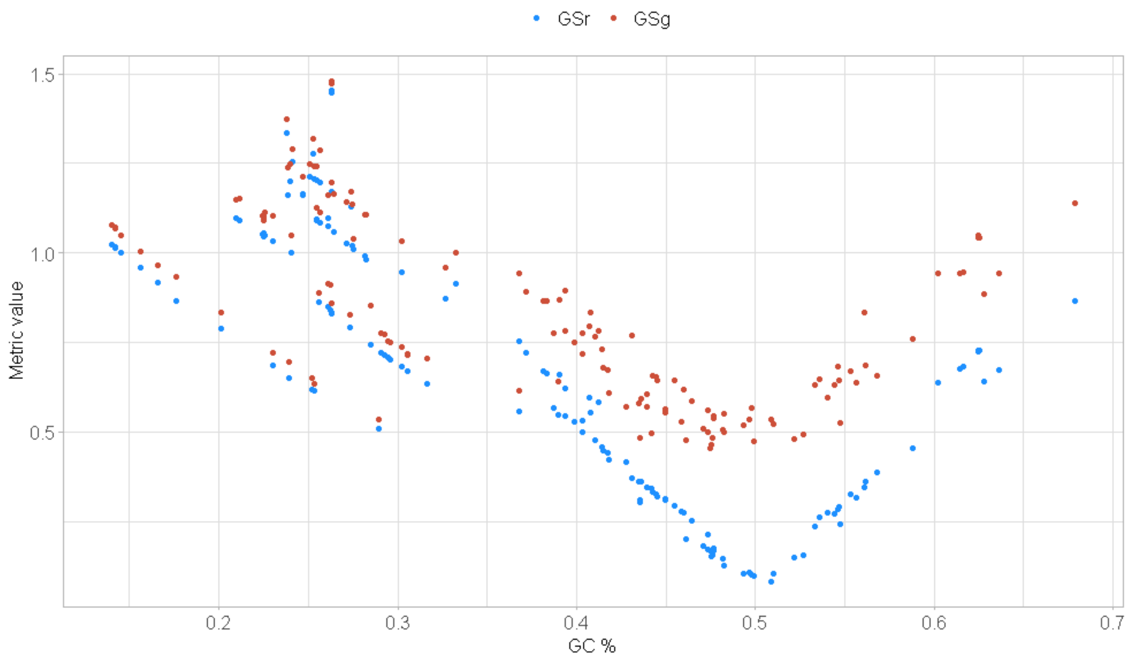

Supplementary Figure S3. Representation of the Genome Signature measure of the provided genome (GS_g) and a random genome of its same length (GS_r) against GC content. The k-values selected for each genome are those where the maximum is found for GS_p (GS_g - GS_r) between $k = 2$ and $k = 16$.

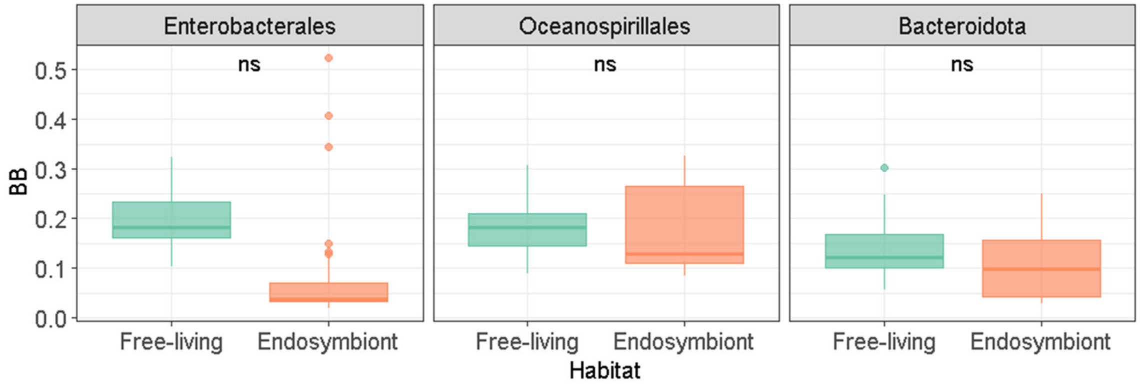

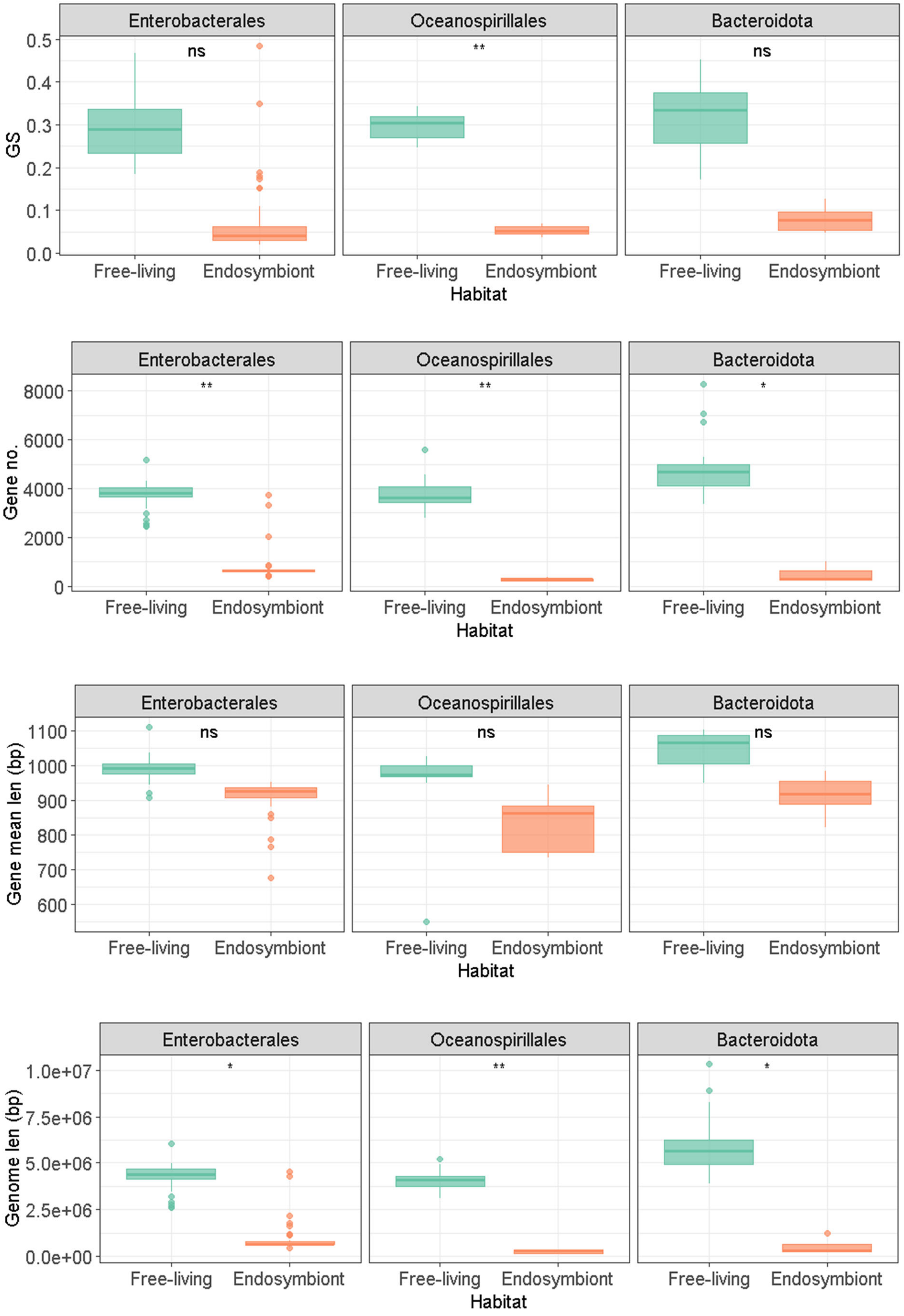
Enterobacterales
Oceanospirillales
Bacteroidota
ns
**
ns
GS
0.0
0.1
0.2
0.3
0.4
0.5
Free-living
Endosymbiont
Habitat
Enterobacterales
Oceanospirillales
Bacteroidota
**
**
*
Gene no.
0
2000
4000
6000
8000
Free-living
Endosymbiont
Habitat
Enterobacterales
Oceanospirillales
Bacteroidota
ns
ns
ns
Gene mean len (bp)
600
700
800
900
1000
1100
Free-living
Endosymbiont
Habitat
Enterobacterales
Oceanospirillales
Bacteroidota
*
**
*
Genome len (bp)
0.0e+00
2.5e+06
5.0e+06
7.5e+06
1.0e+07
Free-living
Endosymbiont
Habitat

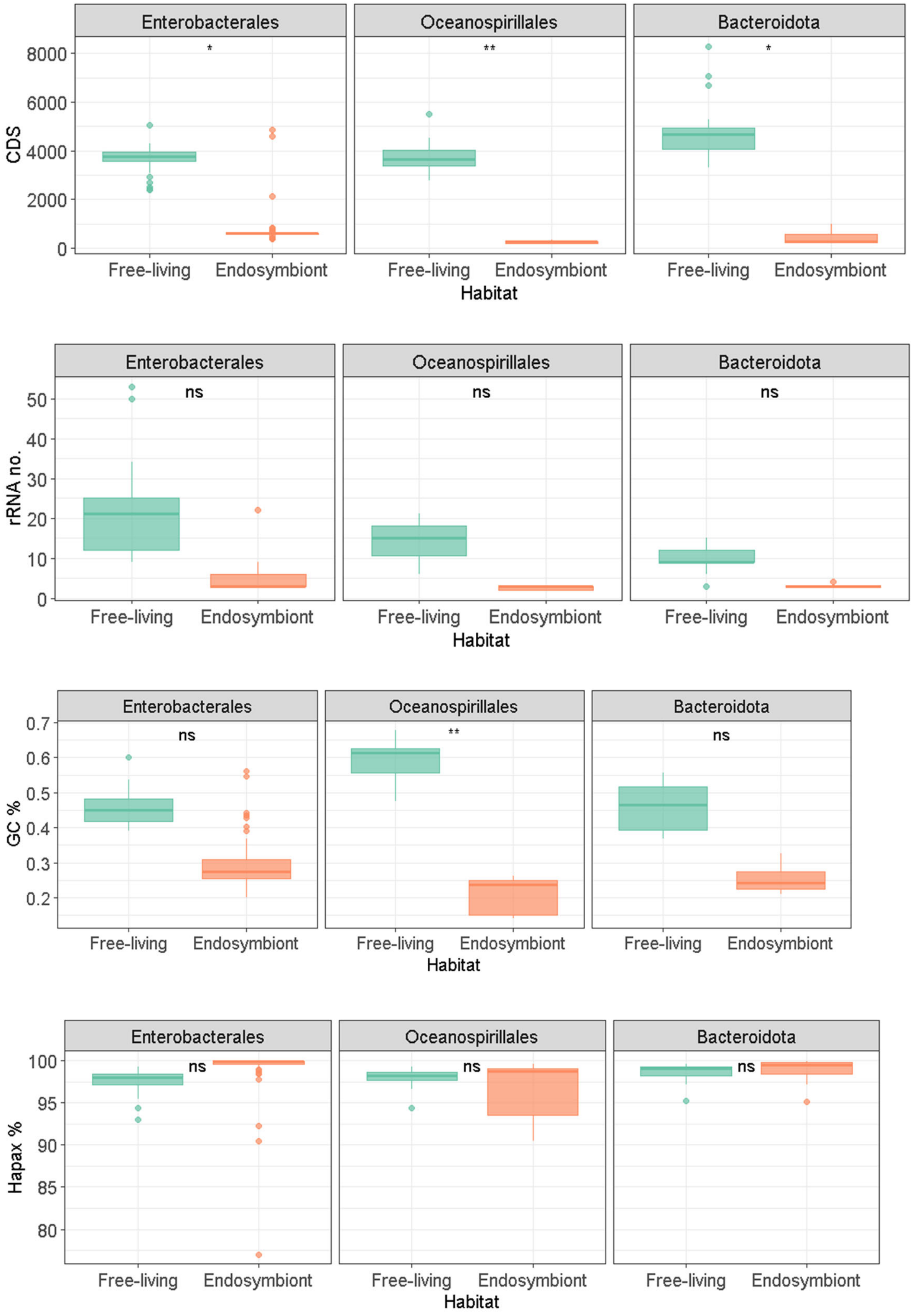

Supplementary Figure S4. Boxplots of each trait for free living and endosymbiont genomes in each studied clade. In stars, we show the statistical significance of the mean based on the phylogenetically informed test.

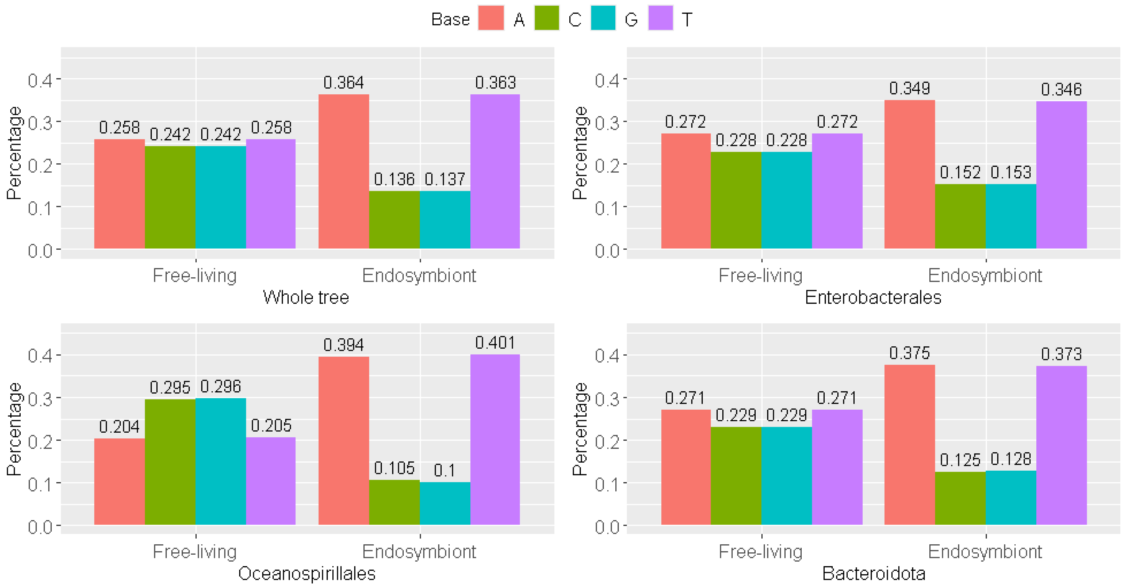

Supplementary Figure S5. Barplots of the mean base composition of the genomes for each habitat and within each group.
